# A multi-sensory stimulating attention model for cities’ taxi service demand prediction

**DOI:** 10.1038/s41598-022-07072-z

**Published:** 2022-02-23

**Authors:** Lyuchao Liao, Yongqiang Wang, Fumin Zou, Shuoben Bi, Jinya Su, Qi Sun

**Affiliations:** 1 Fujian Provincial Universities Key Laboratory of Industrial Control and Data Analysis, Fujian University of Technology, Fuzhou, 350118 China; 2 Fujian Key Laboratory of Automotive Electronics and Electric Drive, Fujian University of Technology, Fuzhou, 350118 China; 3 School of Geographical Science, Nanjing University of Information Science and Technology, Nanjing, 210044 China; 4 School of Computer Science and Electronic Engineering, University of Essex, Colchester, CO4 3SQ UK; 5 School of Vehicle and Mobility, Tsinghua University, Beijing, 100084 China

**Keywords:** Information technology, Computer science

## Abstract

Taxi demand forecasting is crucial to building an efficient transportation system in a smart city. Accurate taxi demand forecasting could help the taxi management platform to allocate taxi resources in advance, alleviate traffic congestion, and reduce passenger waiting time. Thus, more efforts in industrial and academic circles have been directed towards the cities’ taxi service demand prediction (CTSDP). However, the complex nonlinear spatio-temporal relationship in demand data makes it challenging to construct an accurate forecasting model. There remain challenges in perceiving the micro spatial characteristics and the macro periodicity characteristics from cities’ taxi service demand data. What’s more, the existing methods are significantly insufficient for exploring the potential multi-time patterns from these demand data. To meet the above challenges, and also stimulated by the human perception mechanism, we propose a Multi-Sensory Stimulus Attention (MSSA) model for CTSDP. Specifically, the MSSA model integrates a detail perception attention and a stimulus variety attention for capturing the micro and macro characteristics from massive historical demand data, respectively. The multiple time resolution modules are employed to capture multiple potential spatio-temporal periodic features from massive historical demand data. Extensive experiments on the yellow taxi trip records data in Manhattan show that the MSSA model outperforms the state-of-the-art baselines.

## Introduction

A smart city is as considered the direction of urban development and the trend of civilized development in the information age^1^. It aims to use modern information technology to promote the interconnection, efficiency, and intelligence of urban operating systems^2^. As an indispensable part of a smart city, intelligent transportation aims to improve the transportation system's operating efficiency and make full use of transportation resources^3^. Taxi is a travel tool that meets people's travel service demand, and it plays an essential role in urban public transportation systems^4^. In recent years, with the continuous improvement of people's living standards and the significant lifestyle changes, there is higher requirements for quality and efficient taxi services. However, there are still some inefficiencies in this mode of transportation. For example, the insufficient supply of taxis in some areas leads to long waiting times for passengers, and the oversupply in some areas causes a large number of online taxis to be empty. These problems not only lead to inefficient taxi cruising, but also affect people's satisfaction with taxi services. Therefore, to better meet the needs of passengers and reduce costs, the taxi management platform should conduct reasonable dispatch of online taxis. At present, with the increasing popularity of floating car technology and taxi services like Uber and Didi Chuxing, a massive amount of public travel data has been accumulated^5^. Making full use of these historical public travel data provides us an opportunity to address the challenges of CTSDP in smart cities^4^, which could rationally dispatch taxis to areas with higher demand and reduce the waiting time of passengers. In addition, it could also reduce energy consumption and air pollution in smart cities. In summary, building an efficient CTSDP model for the massive historical demand data is crucial to constructing a smart traveling service in smart cities.

More efforts have been directed to towards building a more accurate model with historical data for traffic prediction. Data-driven traffic prediction methods are generally divided into three major categories: time-series analysis methods, traditional machine learning methods, and deep learning based methods. The time-series analysis methods and the traditional machine learning methods are insufficient for the complex nonlinear temporal and spatial relationships in traffic data. Fortunately, deep learning has brought new ideas to capture nonlinear spatio-temporal relationships^6–8^, which has aroused more and more related studies. Abundant studies have applied Convolutional Neural Network (CNN) to capture spatial correlations^9–11^, and Zhang et al.^12^ further proposed ST-ResNet, which is composed of a convolutional layer and a residual unit to simulate the spatial dependence of the city. To capture the time dependence, recurrent neural networks (RNN) and some of its variants, such as long short-term memory networks (LSTM) and gated recurrent units (GRU), have been widely used due to their excellent performance in capturing dynamic time dependence^6,13,14^. For example, Xu et al.^15^ encoded the taxi demand data in the past week into a time series and fed the sequence to the LSTM network to learn the time pattern of taxi demand. Although these studies explicitly modeled temporal or spatial dependence, they don’t consider both aspects simultaneously. Therefore, to solve the problem of spatio-temporal sequence prediction, Liu et al.^16^ further proposed a spatio-temporal network for demand prediction, which integrates local CNN and LSTM networks to learn spatio-temporal correlations simultaneously. Wang et al.^17^ also used an encoder-decoder framework to deal with the spatio-temporal relationships of traffic data. Taking into account the shortcomings of a single data source, Zhao et al.^18^ proposed a feature fusion model using multi-source data for prediction. It verified the effectiveness of the model using multi-source data sets. Some studies have extended the spatio-temporal model to solve CTSDP which requires higher accuracy. For example, Rodrigues et al.^19^ proposed a deep learning architecture combining text information and time-series data and applied this method to regional taxi demand prediction.

The existing methods mainly focus on predicting taxi demand in some specific regions. There are few studies which are based on the taxi Origin–Destination (OD) demand in a whole city. One of the significant challenges in CTSDP is to capture the spatial–temporal dependencies between each two OD pairs. Liu et al.^20^ proposed a contextualized spatio-temporal network for taxi Origin–Destination demand prediction. The network integrates local spatial context, temporal evolution context, and global context into a framework. Ke et al.^21^ proposed the spatio-temporal encoder-decoder residual multi-graph convolutional network (ST-ED-RMGC), which builds multiple graphs to characterize the non-Euclidean pair-wise geographical and semantic correlations among different OD pairs. Chen et al.^22^ proposed a method combining spatial-OD and Bidirectional ConvLSTM model with taking the historical and future states of the data into account to extract the time and space characteristics. Although these spatio-temporal deep networks showed reasonable performance for CTSDP, they still show some significant shortcomings: (1) being insufficient to perceive the micro spatial characteristics and the macro periodicity characteristics from the cities’ taxi service demand data; (2) lack of consideration for exploring the potential multi-time patterns from these demand data.

In the human perception mechanism, people generally pay attention to the changes in details and the macro variety tendency^23^. Therefore, to meet the above challenges, we propose a Multi-Sensory Stimulus Attention (MSSA) model for CTSDP, which is similar to the multi-sensory stimulus in the human perception mechanism^24^. Specifically, the proposed framework integrates two different attention mechanisms, namely Detail Perception Attention (DPAtt) and Stimulus Variety Attention (SVAtt), for mining the characteristics of historical demand data at the micro and macro levels respectively. Moreover, the framework combines a Local Spatio-temporal Network (LSTN), a Daily periodicity network (DayNet), and a Weekly periodicity network (WeekNet) for exploring potential multiple periodic patterns.

The main contribution of this work could be summarized as follows:

- Inspired by the human perception mechanism, we propose a Multi-Sensory Stimulus Attention (MSSA) model, which combines detail perception attention and stimulating variety attention to learn the characteristics of historical demand data from the macro and micro levels.
- We combine multiple time resolution modules to capture potential spatio-temporal periodicity features from massive taxi service demand data.
- Extensive experiments were conducted on Manhattan's yellow taxi trip records data, and the results show that the proposed MSSA model outperforms the state-of-the-art methods.

## Results and discussion

### Prediction results and overall performance analysis

We compare the prediction performance of the proposed method with the following baseline methods. These baseline methods include not only time-series analysis methods and traditional machine learning methods but also the state-of-the-art deep learning models. The specific methods are:

- *Historical Average (HA)* Historical Average uses the average values of previous demand at a given location over a relative time interval to forecast demand.
- *Linear Regression* We implemented two typical methods: **Lasso**^25^ (i.e., with $l_{1}$-norm regularization) and Ordinary Least Squares Regression^26^ (**OLSR**).
- *Multilayer Perceptron (MLP)* The multilayer perceptron consists of four fully connected layers. The MLP takes the demand data of the last n time intervals as input and predicts the demand data of the next moment.
- *XGBoost*^27^ XGBoost is a robust boosting tree-based method that is widely used in data mining applications.
- *ST-ResNet*^28^ ST-ResNet is a deep learning-based traffic prediction method, which constructs traffic maps of cities at different times in the form of images.
- *CSTN*^20^ CSTN is the state-of-the-art deep learning-based method for taxi Origin and Destination demand prediction. The method is modeled from three perspectives: local spatial context, temporal evolution context, and global relevance context.

We evaluate the prediction performance of our proposed method and existing methods with two widely used metrics, namely Mean Average Percentage Error (MAPE) and Root Mean Square Error (RMSE), which are defined as:

1 $$MAPE = \frac{{{{100{\%}}}}}{m}\mathop \sum \limits_{t = 1}^{m} \frac{{\left| {\hat{R}\left( t \right) - R\left( t \right)} \right|}}{R\left( t \right)}$$

2 $$RMSE = \sqrt {\frac{1}{m}\mathop \sum \limits_{t = 1}^{m} \left| {\hat{R}\left( t \right) - R\left( t \right)} \right|^{2} }$$

where *m* is the total number of grid divisions in New York City, $\hat{R}\left( t \right)$ and $R\left( t \right)$ are the predicted taxi demand and the corresponding ground truth in time interval $t,$ respectively. These two metrics are the most commonly used to evaluate the prediction accuracy, and their value ranges are $\left[ {0, + \infty } \right)$. A higher value of the metric indicates a higher prediction error.

In our work, we respectively predicted the Origin–Destination demand of taxis and the Origin demand of taxis. When evaluating the performance of these two aspects, for convenience, the above two evaluation metrics were denoted as MAPE-OD and RMSE-OD in the taxi Origin–Destination demand assessment, and MAPE-O and RMSE-O in the taxi Origin demand assessment.

Using MAPE and RMSE evaluation metrics, we summarize the experimental results of all methods in Origin demand forecasting and Origin–Destination demand forecasting in Table 1.

**Table 1:** Performance comparison of methods.

Method	MAPE-O (%)	RMSE-O	MAPE-OD (%)	RMSE-OD
HA	45.04	52.44	37.71	1.93
Lasso	34.89	33.00	33.85	1.65
OLSR	33.09	32.68	33.86	1.65
XGBoost	37.78	31.23	32.04	1.54
MLP	25.24	25.60	30.70	1.49
ST-ResNet	24.16	22.43	28.53	1.38
CSTN	18.48	19.85	27.37	1.32
MSSA	15.10	14.36	25.93	1.25

The experimental results could be observed that the MSSA model significantly outperforms other competed methods. Among them, the time-series analysis method (HA) offers the worst performance. The traditional machine learning methods (Lasso, OLSR, and XGBoost) are relatively better than the former, and the methods based on deep learning (ST-ResNet and CSTN) have more significant improvements. Specifically, the MSSA method our proposed achieves the lowest MAPE and RMSE on the task of taxi Origin demand prediction and taxi Origin–Destination demand prediction. Moreover, for the Origin demand prediction, the MSSA model achieves 18.29% and 27.66% relative performance improvements over MAPE-O and RMSE-O, compared to the existing best-performing CSTN method. For the Origin–Destination demand prediction, the MAPE-OD and RMSE-OD are also reduced by 5.26% and 5.30% respectively compared to CSTN method.

The time-series forecasting methods (HA) does not perform well because it only relies on historical records and ignores spatial and other contextual features. The traditional machine learning methods (Lasso, OLSR, and XGBoost) further treat spatial correlation as features and rules, which helps to obtain better performance gains over the traditional time series analysis methods. However, these methods generally fail to capture the complex and nonlinear spatial and temporal correlations. The emerging deep learning methods (ST-ResNet and CSTN) showed significant improvement in recent years. However, there are still some drawbacks in the ST-ResNet and CSTN, which lead to the limitations of further improvement. Generally, ST-ResNet ignores learning the temporal evolution context, and CSTN ignores the influence of an obvious pattern (periodicity). In addition, these methods are not sufficient to perceive the micro spatial characteristics and the macro periodicity characteristics from the cities’ taxi service demand data. Compared with these methods, the MSSA model further integrates detail perception attention and stimulating variety attention to learn the characteristics of historical demand data at the macro and micro levels and then combines multiple time resolution modules to capture various potential spatio-temporal periodicity features.

### Performance analysis on different days

**Figure 1:**
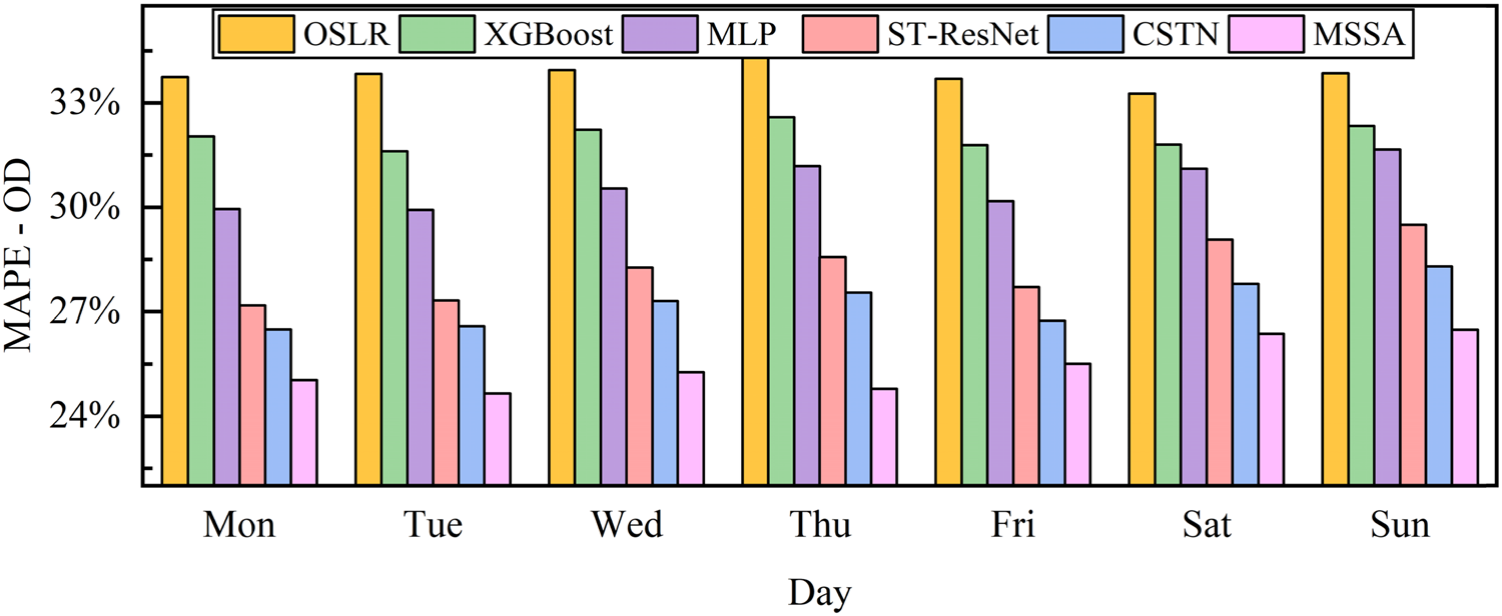
Performance comparison on different days of the week.

We could compare the prediction performance of all methods on different days by dividing the dataset into 7 categories according to different days of the week and predicting separately. As shown in Fig. 1, we only compare the performance of traditional machine learning methods and deep learning methods on the metric MAPE-OD because the performance of time series methods is poor and the performance comparison of other metrics is similar to MAPE-OD. We can conclude from the figure that our proposed method outperforms all baseline methods on different days of the week. In addition, the model could achieve lower MAPE-OD values on weekdays than on weekends. We can also conclude that the prediction performance of weekdays is better than that of weekends. The underlying reasons is that the taxi trips on weekdays are more regular, which is beneficial to the model prediction, while the trips on weekends are more random, which increases the difficulty of the model's prediction.

### Effectiveness of multi-sensory stimulating attention

This section discusses the impact of multi-sensory stimulating attention on the performance of taxi demand prediction. We trained a network without multi-sensory stimulating attention and got the average of its experimental results through multiple training. The comparison results are shown in Table 2.

**Table 2:** Performance comparison for detail perception attention.

Method	MAPE-O (%)	RMSE-O	MAPE-OD (%)	RMSE-OD
MSS	16.98	15.29	26.67	1.274
MSSA	15.10	14.36	25.93	1.258

The comparison results show that the MSS model without multi-sensory stimulus attention achieves a MAPE-O of 16.98% and a RMSE-O of 15.29, and it also gets a MAPE-OD of 26.67% and a RMSE-OD of 1.274. After adding the multi-sensory stimulating attention, the performance of the MSSA model with the multi-sensory stimulus attention will be improved with 11.07% and 6.08% over metrics MAPE-O and RMSE-O respectively. It also will be improved with 2.77% and 1.26% over metrics MAPE-OD and RMSE-OD respectively. The experimental results show that the multi-sensory stimulus attention could improve the model's prediction performance.

### Influence of sequence length

**Figure 2:**
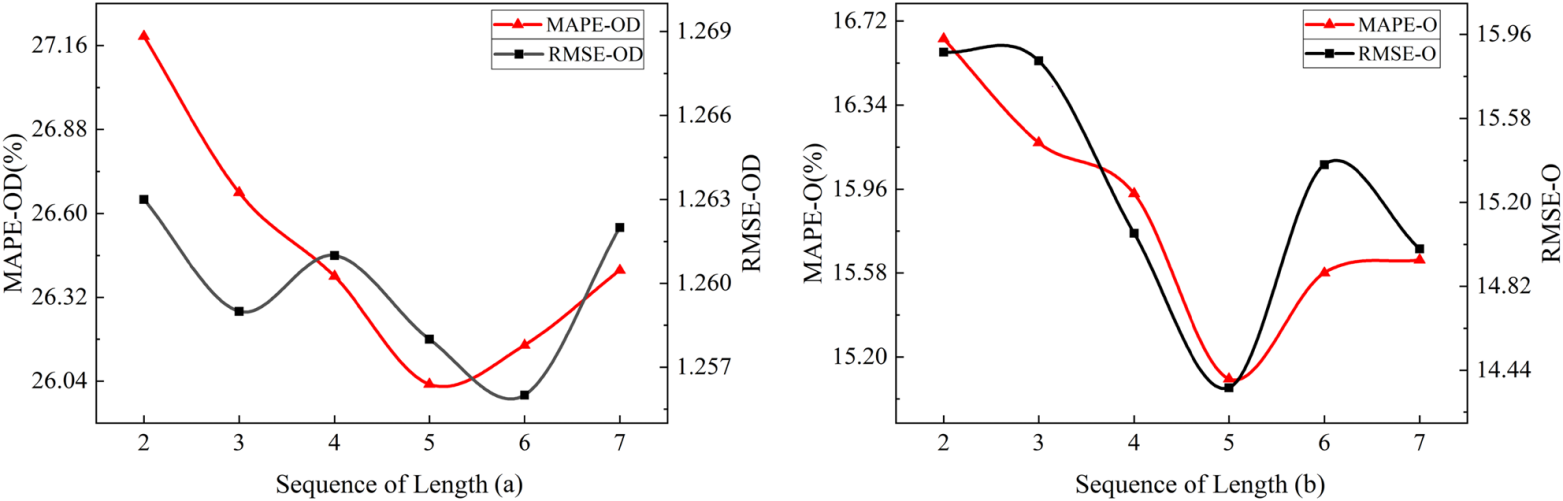
Relationship analysis for between different sequence lengths.

To explore the influence of sequence length in the LSTN network, we investigate the relationship between sequence length and prediction performance by training our model with sequences of different lengths. The relationship between the sequence length and the Origin demand prediction performance is shown in Fig. 2a, and the relationship between the sequence length and the Origin–Destination demand prediction performance is shown in Fig. 2b.

As shown in Fig. 1, the prediction performance of model shows an increasing tendency with the increase of sequence length (the lower the value of the evaluation metric, the better). The sequence length offers an optimal value at the length of 5 (2.5 h). It’s noteworthy that the prediction performance begins to decline when exceeding the optimal value. The upward trend of the prediction performance with increasing sequence length indicates that the model is sensitive to the variety tendency in the same period, and the decline when exceeding the optimal value means that more parameters need to be learned when extending to a more extensive time range. The sequence length was set to 5 in the MSSA model to make the training convergence more quickly.

### Effect with different periodic modules

To further explore the impact of different potential periodic modules on the overall performance, we integrate different periodic modules to implement the following variants:

- *LSTN + DayNet* The network only contains local spatio-temporal modules and potential daily periodic modules. The outputs of the two modules were fused and fed into a convolution layer for predicting taxi demand.
- *LSTN + WeekNet* The network contains only a local spatio-temporal module and a potential weekly periodicity module. The outputs of these two modules were fused and fed into a convolutional layer for predicting cab demand.
- *LSTN + DayNet + WeekNet* As a complete version of our proposed model, this network incorporates a local spatio-temporal module, a potential daily periodicity module, and a potential weekly periodicity module to predict taxi demand.

The prediction performance of different variants on taxi demand is shown in Table 3. The comparative analysis shows that both the daily and the weekly periodic modules are beneficial for prediction performance, and the integrating multiple periodicity modules shows better prediction performance.

**Table 3:** Comparison of different periodicity modules.

Method	MAPE-O (%)	RMSE-O	MAPE-OD (%)	RMSE-OD
LSTN + DayNet	17.78	15.21	26.24	1.271
LSTN + WeekNet	16.99	15.03	26.19	1.269
LSTN + DayNet + WeekNet	15.10	14.36	25.93	1.258

## Methods

### Taxi trip records data

The experiments were conducted on the taxi travel record dataset provided by NYC open data^29^, which is mainly distributed in Manhattan. We selected the taxi travel record data for the whole year of 2014. The data of the last 60 days was employed as the test set, and the rest was used as the training set.

**Figure 3:**
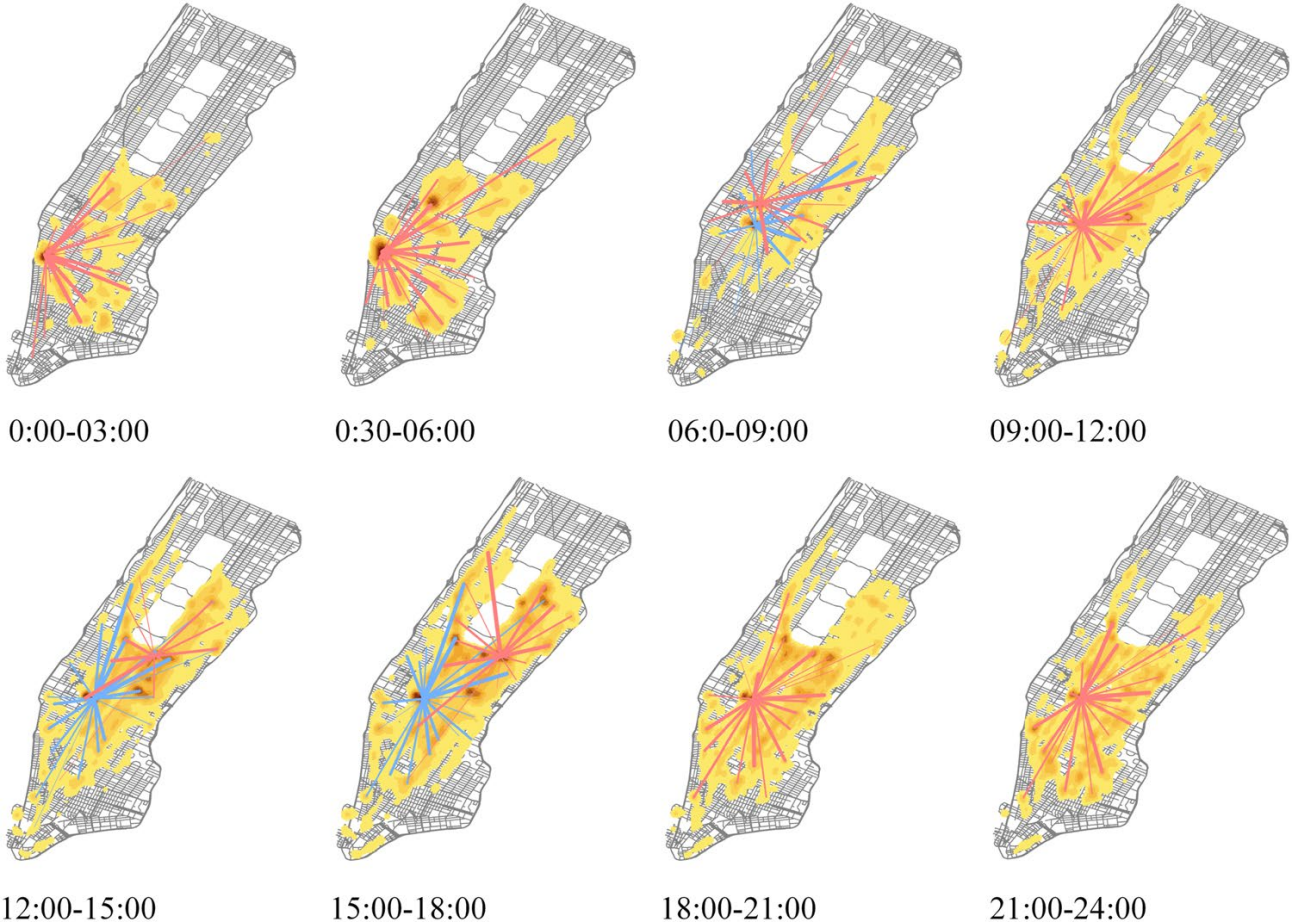
Travel heat map in different time frame. These eight maps were created using ArcMap version 10.5 software (https://desktop.arcgis.com/).

In addition, we analyzed the temporal and spatial characteristics of urban residents' travel. We firstly divided the travel records on January 10, 2014 into 8 time periods and then drew the travel heat map of each time frame. In the heat map, the darker color indicates that there is a high demand for taxis in this area, and different colored lines represent popular travel routes from different high-demand areas. From the results shown in Fig. 3, it could be learnt that the cities own different demand of taxi service in different time period. It’s a challenge to capture these periodical patterns from massive taxi service demand data, and we aim to address this challenge in this work.

### Data enhancement

To achieve data enhancement through multi-source information fusion, we collected meteorological data from open-source websites^30^. The collected and selected meteorological indicators are shown in Table 4, including 23 discrete weather types and 6 continuous weather indicators.

**Table 4:** Types of meteorological data in Manhattan.

Type	Information
Weather Condition	23 types(e.g., Sunny, Rainy)
Temperature/℃	[− 18.3, 35.6]
Windchill/℃	[− 28.4, 38.5]
Visibility/km	[0.4, 16.1]
Wind Speed/km/h	[0.0, 137.0]
Humidity/%	[9, 100]
Precipitation/mm	[0.0, 28.7]

### Data preprocessing

**Figure 4:**
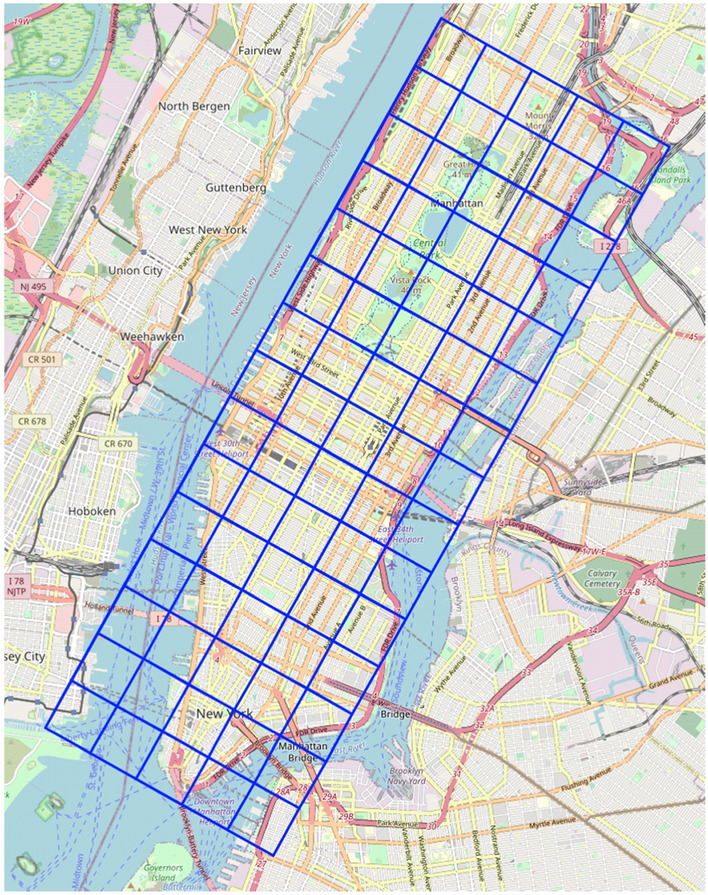
Grid delineation map of the study area. This map was created by using a third-party package of python, named Folium (https://python-visualization.github.io/folium/), and the version number of the package is 0.12.1. The base map for this figure was produced by OpenStreetMap. Credit: OpenStreeMap contributors. This map is licensed under Open Database License. The license terms can be found at the following link: https://wiki.osmfoundation.org/wiki/Licence.

In the spatial dimension, we divide the Manhattan area into H × W grid areas, as shown in Fig. 4, and numbered them as $\left\{ {g_{1} ,g_{2} , \ldots ,g_{G} } \right\}$, where ${\text{G}} = {\text{H}} \times {\text{W}}$. In the time dimension, we divided the time evenly into a sequence of time intervals, denoted as $\left\{ {t_{1} ,t_{2} , \ldots ,t_{T} } \right\}$. Based on the time stamp and location coordinates of passengers getting on and off the taxi from the taxi trip record, we counted the number of trips between each area and then generate the taxi OD matrix $R\left( t \right)$ within the time interval t. We denoted it as $R\left( t \right) \in R^{G \times G}$.

To better maintain the continuity of data distribution and better capture local spatial features, we further carried out a simple transformation of the dimension of the matrix. That is, transformed from the original $R\left( t \right) \in R^{G \times G}$ to $R\left( t \right) \in R^{G \times H \times W}$. In addition, we filtered the samples with demand values less than 10 to improve data quality^31^.

For the meteorological data, we first digitized the 23 discrete types of data with one-hot encoding and then converted the 6 continuous types of data into the range of [−1,1] with Min–Max linear normalization.

### Model framework overview

**Figure 5:**
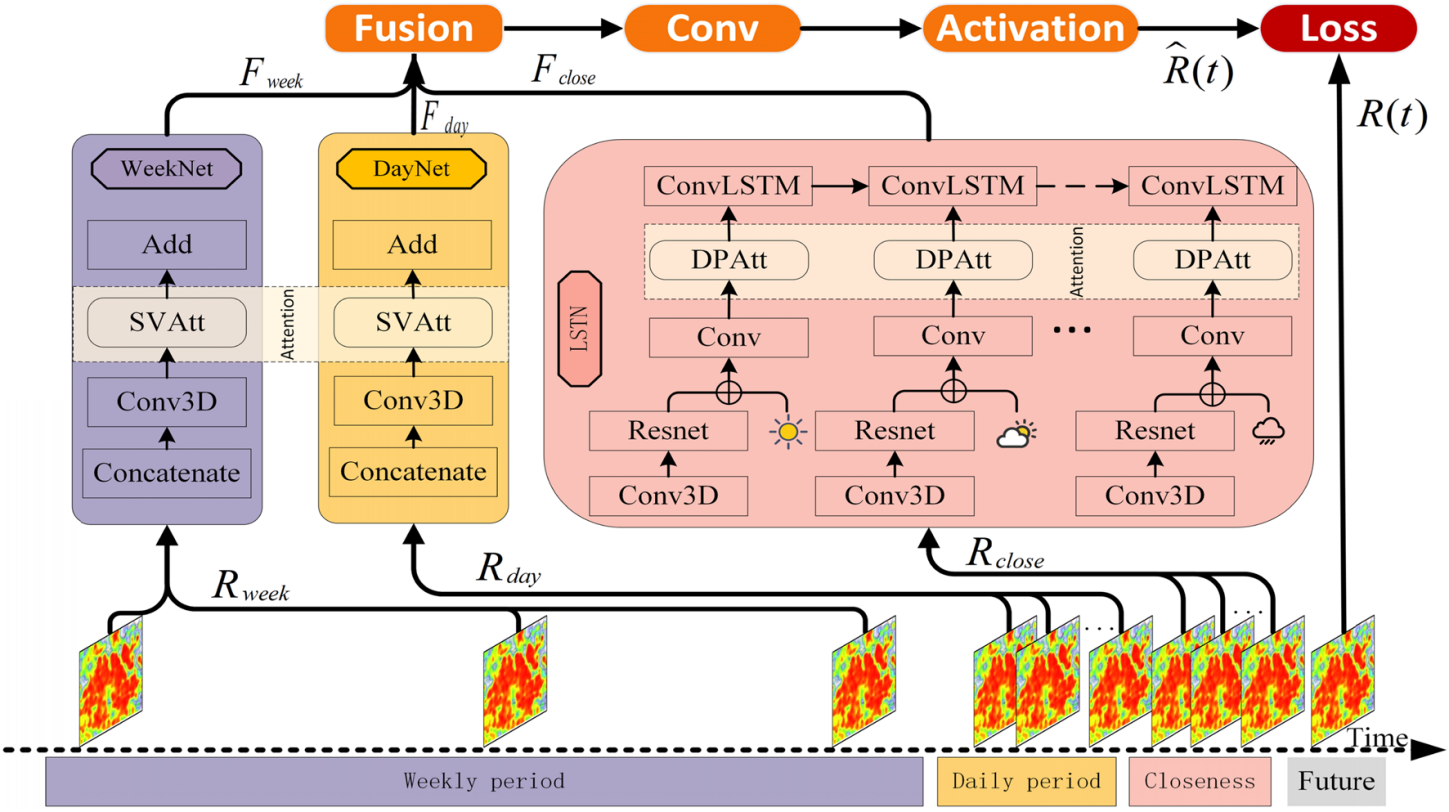
The system architecture of MSSA.

This section provides details about the MSSA model, which aims to predict the Cities’ Taxi Service demand $\hat{R}\left( t \right)$ for possible upcoming trips by using nearby historical demand data ${\text{R}}_{close}$, daily periodic data $R_{day}$, weekly periodic data $R_{week}$, and the heterogeneous data $\epsilon$. Figure 5 shows the architecture of the MSSA model, which consists of three sub-networks, namely LSTN, DayNet, and WeekNet. These three sub-networks separately model different periods of historical demand data to analyze the three time modes: closeness, daily periodicity, and weekly periodicity. What's more, the Detail Perception Attention (DPAtt) learns the spatial and meteorological characteristics of historical demand data at the micro-level, and the Stimulating Variety Attention (SVAtt) learns potential periodic patterns from the macro level.

### Local spatial feature learning

In spatio-temporal traffic data, the traffic observations at spatially adjacent locations are often not independent but are strongly correlated. The same is true for taxi demand, which tends to have more similar demand patterns in spatially adjacent regions. To extract local spatial features of taxi demand from the Origin view and the Destination view, we input the taxi OD demand matrix $R\left( t \right)$ and DO demand matrix $R\left( t \right)^{T}$ into the 3D convolutional layer, respectively, which has 16 convolutional kernels with the kernel size of 3 × 3. Formally,

3 $$F^{o} = Conv\left( {R\left( t \right),w^{o} } \right)$$

4 $$F^{d} = Conv\left( {R\left( t \right)^{T} ,w^{d} } \right)$$

where $w^{o}$ and $w^{d}$ are learnable parameters.

The residual learning proposed by He et al.^32^ was applied in our model to further explore detailed spatial features with limited computing resources. In our work, the residual mapping consists of the combination of activation and convolution once. We stacked *L* residual units upon Conv3D as follows:

5 $$F^{{o,\left( {l + 1} \right)}} = F^{o,\left( l \right)} + \xi \left( {F^{o,\left( l \right)} ,\theta^{o,\left( l \right)} } \right),l = 1,2, \ldots ,L$$

6 $$F^{{d,\left( {l + 1} \right)}} = F^{d,\left( l \right)} + \xi \left( {F^{d,\left( l \right)} ,\theta^{d,\left( l \right)} } \right),l = 1,2, \ldots ,L$$

where *ξ* is the residual function, and $\theta^{o,\left( l \right)} ,\theta^{d,\left( l \right)}$ are the set of all learnable parameters of the $l$th residual unit in the Origin view and the Destination view, respectively.

After extracting the local space features of the Origin and the Destination view, we finally use a convolutional layer to fuse the two features. Formally,

7 $$F^{od} = Conv\left( {F^{{o,\left( {L + 1} \right)}} { }F^{{d,\left( {L + 1} \right)}} ,w_{od} } \right)$$

where ⊕ denotes feature concatenation operation and $w_{od}$ is the parameter of the fused convolutional layer.

Since our task is to predict the future demand of taxis in each city grid, the network structure didn’t employ any downsampling and pooling operations in the spatial dimension. This is because these operations tend to reduce the size of the tensor which makes the network less sensitive to local spatial features.

### Heterogeneous information fusion

Taxi demand may be affected by complex external factors like road network connections, points of interest, and weather conditions. Considering the potential strong correlation of meteorological information on taxi trips, we focused on the impact of meteorological data. We first encode them using a multilayer perceptron (MLP). Then, we concatenate the encoded meteorological features with the local spatial features and fused the two features with a convolutional layer with 32 filters. Formally,

8 $$F^{odw} = Conv\left( {F^{od} \oplus F^{w} ,w_{odw} } \right)$$

where ⊕ is the feature concatenation. $F^{w}$ denotes the meteorological feature after coding and $w_{odw}$ denotes the parameters of the convolutional layer.

### Detail perception attention

Recently, attention mechanism has become an essential part of neural network structure and has a large amount of research in different fields. In particular, Woo S et al.^33^ proposed a convolutional block attention module (CBAM) to enhance feature representation for visual semantic analysis.

Inspired by the attention in CBAM, we proposed a Detail Perception Attention (DPAtt) to learn the detail variation of fused features fully. To enhance the feature representation of local spatial features and meteorological features, DPAtt is constructed with a level attention module, and a spatial attention module, in which the level attention module mainly processes the feature map of different taxi demand levels and the spatial attention module mainly reinforces the demand density distribution on the feature maps. The level attention and spatial attention in DPAtt make it possible to pay more attention to the vital demand levels and the critical regions in the feature maps.

**Figure 6:**
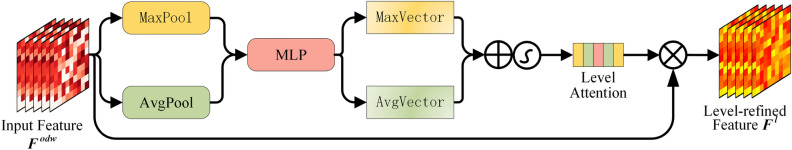
The level attention.

As shown in Fig. 6, the level attention does max-pooling and average-pooling for the feature maps in level dimension to obtain Maxpool level attention vector and AvgPool level attention vector. We then input these two vectors into a single-layer perceptron with shared weights to obtain two new level attention vectors. We merged these two vectors using element-wise summation and finally multiplied with the original feature map to obtain the new feature map, which is expressed as:

9 $$M_{l} \left( {F^{odw} } \right) = {\upsigma }\left( {{\text{MLP}}\left( {MaxPool\left( {F^{odw} } \right)} \right) + {\text{MLP}}\left( {AvgPool\left( {F^{odw} } \right)} \right)} \right)$$

10 $$F^{l} = M_{l} \left( {F^{odw} } \right) \otimes F^{odw}$$

where ⊗ denotes the multiply operation, and $\sigma$ denotes the sigmoid function.

**Figure 7:**
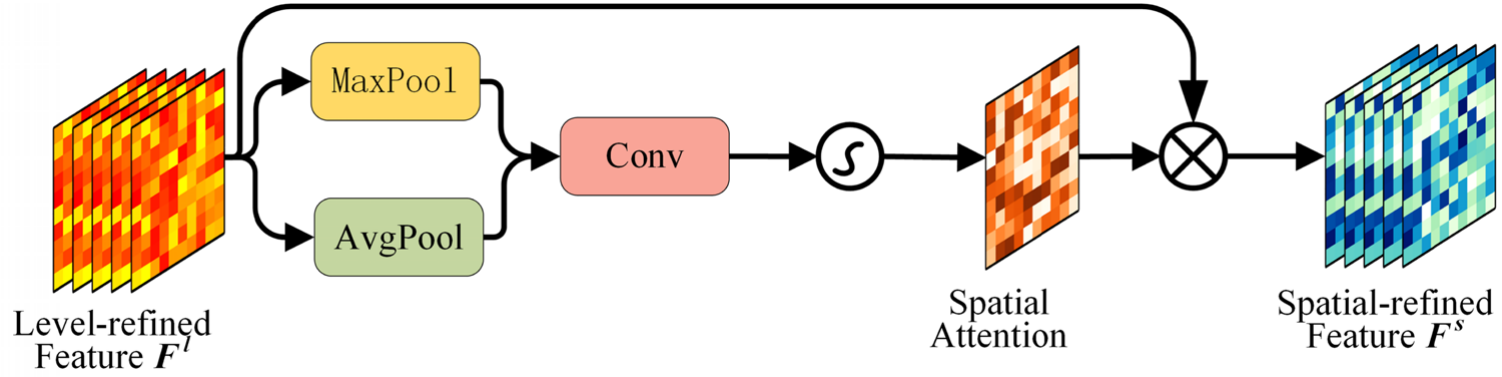
The spatial attention.

To compute the spatial attention, as shown in Fig. 7, we first applied average-pooling and max-pooling operations along the level axis to obtain two spatial attention maps and concatenate them. Afterwards, a convolutional layer and an activation layer were employed to generate the spatial attention weight matrix. Finally, we multiplied the matrix with the input feature map to obtain the weighted feature matrix, which is expressed as:

11 $$M_{s} \left( {F^{l} } \right) = \sigma \left( {Conv\left( {\left[ {MaxPool\left( {F^{l} } \right);AvgPool\left( {F^{l} } \right)} \right]} \right)} \right)$$

12 $$F^{s} = M_{s} \left( {F^{l} } \right) \otimes F^{l}$$

where ⊗ denotes the multiply operation, and $\sigma$ represents the sigmoid function.

### Local temporal feature learning

To simultaneously extract time closeness features and spatial features of taxi demand, we introduced the ConvLSTM^34^ network, which helps establish time-series relationships from two-dimensional plane data and extract spatial relationships. The difference with LSTM is that the convolution is employed as the operation between the input and each gate, which helps to extract both temporal features and spatial features.

The operation could be formulated as follows:

13 $$\begin{aligned} i_{t} & = \sigma \left( {w_{xi} {*}X_{t} + w_{hi} {*}h_{t - 1} + w_{ci} \otimes c_{t - 1} + b_{i} } \right) \\ f_{t} & = \sigma \left( {w_{xf} {*}X_{t} + w_{hf} {*}h_{t - 1} + w_{cf} \otimes c_{t - 1} + b_{f} } \right) \\ c_{t} & = f_{t} \otimes c_{t - 1} + i_{t} \otimes \tanh \left( {w_{xc} {*}X_{t} + w_{hc} {*}h_{t - 1} + b_{c} } \right) \\ o_{t} & = \sigma \left( {w_{xo} {*}X_{t} + w_{ho} {*}h_{t - 1} + w_{co} \otimes c_{t} + b_{o} } \right) \\ h_{t} & = o_{t} \otimes {\text{tanh}}\left( {c_{t} } \right) \\ \end{aligned}$$

where $i_{t}$, $f_{t}$ and $o_{t}$ denote input gate, forget gate, and output gate, respectively. $X_{t}$ and $c_{t}$ denote the input and cell state of the network at time *t*, respectively. Each $w$ and $b$ represent the weight and bias of each gate respectively. ${*}$ denotes the convolution operation, $\otimes$ means the Hadamard product and $\sigma$ is the logistic sigmoid function.

We input the temporal closeness subsequence $F_{c}^{s} = \left[ {F^{s} \left( {t - n} \right),F^{s} \left( {t - n + 1} \right), \ldots ,F^{s} \left( {t - 1} \right)} \right]$ into the ConvLSTM network to generate local spatio-temporal features $F_{close}$. Formally,

14 $$F_{close} = ConvLSTM\left( {F_{c}^{s} ,w_{close} } \right)$$

where $w_{close}$ are all learnable parameters.

### Potential temporal periodicity feature learning

Due to the regularity of people's daily life, traffic data usually show apparent cycles and trends. However, most researchers currently model the taxi demand forecasting problem using data from only a few intervals (typically several hours). They ignore a vital property of spatio-temporal prediction: long-term dependence (such as potential periodicity)^35^. Therefore, this section will focus on the learning method of the multi-period mode.

1. Potential daily periodic feature learning

**Figure 8:**
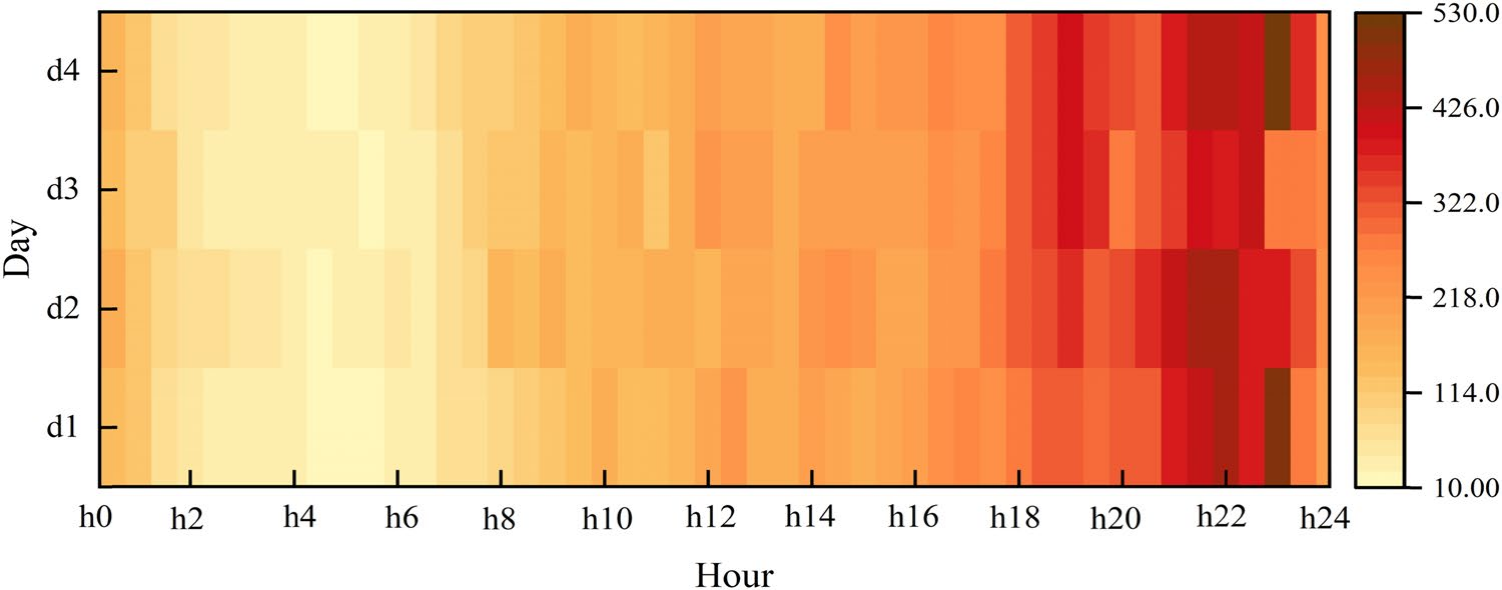
Daily periodicity.

The demand for taxis in the area around times square for four consecutive days is shown in Fig. 8. It could be observed from the figure that there is a significant similarity in demand for taxis at the same time on different days. To learn this potential daily periodic pattern, we denoted the daily relative time interval demand sequence as:

15 $$R_{day} = \left[ {R\left( {t - s_{p} \times p} \right),R\left( {t - \left( {s_{p} - 1} \right) \times p} \right), \ldots ,R\left( {t - p} \right)} \right]$$

where $p$ is the total number of time intervals in a day, and $s_{p}$ is the number of periods of potential daily periodicity.

In the DayNet module, we first concatenate the sequence $R_{day}$ in the time dimension, and then use a 3D convolutional layer to extract features.

16 $$F_{day}^{p} = {\text{Conv}}\left( {R_{day} ,w_{day} } \right)$$

where $w_{day}$ is the learnable parameters.

To perceive which day had the most significant stimulus (i.e., relevance) to our prediction, we further constructed a SVAtt module. (The details of this attention will be expanded in the next section). We input the extracted feature $F_{day}^{p}$ into SVAtt.

17 $$F_{day} = SVAtt\left( {F_{day}^{p} } \right)$$

(2) Potential weekly periodic feature learning

**Figure 9:**
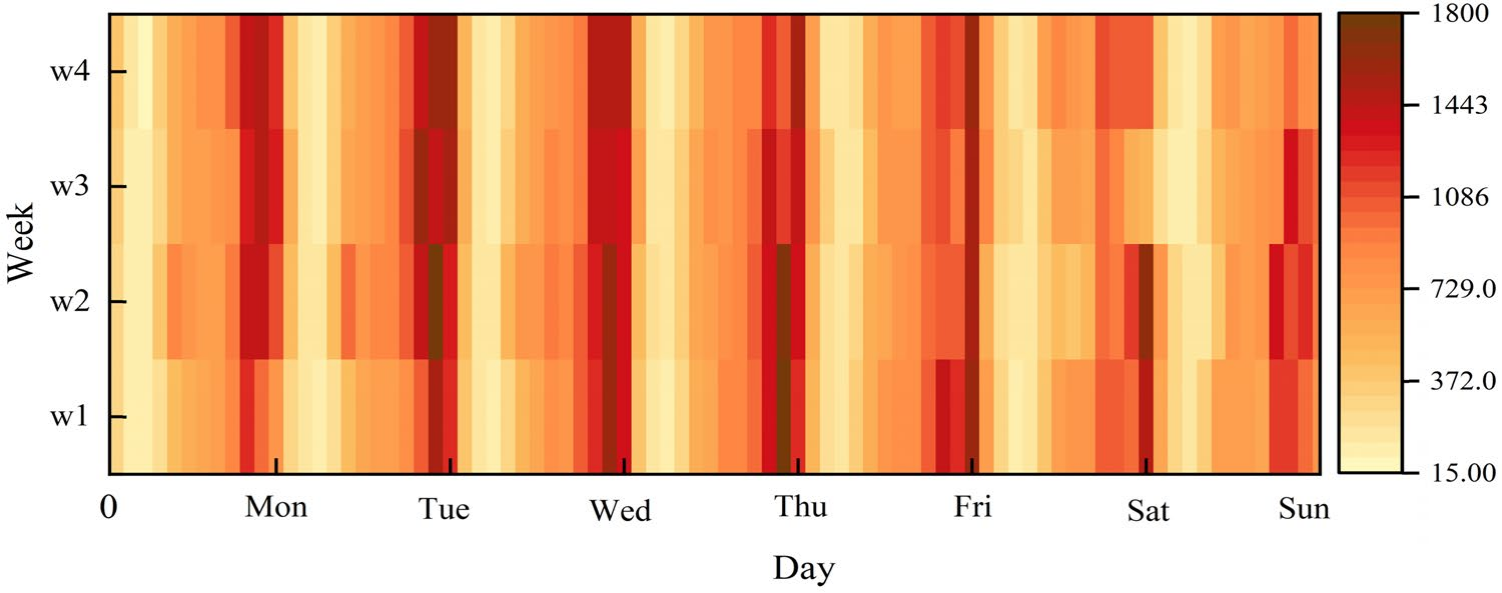
Weekly periodicity.

Similarly, we drew a heat map of the demand situation in the New York times square area for four consecutive weeks, as shown in Fig. 9. From the figure, we can conclude that the taxi demand pattern also has significant similarities at the same time in different weeks. We denoted the weekly relative time interval demand sequence as:

18 $$R_{week} = \left[ {R\left( {t - s_{q} \times q} \right),R\left( {t - \left( {s_{q} - 1} \right) \times q} \right), \ldots ,R\left( {t - q} \right)} \right]$$

where $q$ is the total number of time intervals in a week, and $s_{q}$ is the number of periods of potential weekly periodicity.

In the WeekNet module, we use the same approach as in the DayNet module. That is, convolutional network and SVAtt were used to capture the potential weekly periodic patterns.

19 $$F_{week}^{p} = {\text{Conv}}\left( {R_{week} ,w_{week} } \right)$$

20 $$F_{week} = SVAtt\left( {F_{week}^{p} } \right)$$

where $w_{week}$ is the learnable parameter.

### Stimulating variety attention

In the previous section, when learning the potential daily periodic features, the contribution of the demand of the previous $s_{p}$ days to the forecast was not equal. For example, the impact of the same time for yesterday on the prediction performance may be more significant than that of the day before yesterday, or because of travel restrictions such as car restriction in some areas, people's travel patterns may become more similar on alternate days. Similarly, we believe that similar travel patterns also exist in potential weekly cyclical features. To solve the inconsistent contribution of daily (weekly) data to predictive performance, we propose the SVAtt, which could assign different weights to daily (weekly) demand from two aspects, potential daily periodicity, and potential weekly periodicity.

We detailed each step of the SVAtt module as follows: We first input $F_{day}^{p}$ into a fully connected layer with $s_{p}$ neural units, and then activated it with Softmax to obtain the attention weight vector $w_{day}$.

21 $$W_{day} = Softmax\left( {Liner\left( {F_{day}^{p} } \right)} \right)$$

We further multiplied the $F_{day}^{p}$ with the attention weight $w_{day}$ to obtain the potential daily periodic feature weighted by attention. Formally:

22 $$F_{day} = F_{day}^{p} \otimes w_{day}$$

where ⊗ denotes the Hadamard product.

Similarly, for the potential weekly periodic feature learning module, we use the same method for processing, and the processing process could be expressed as:

23 $$W_{week} = Softmax\left( {Liner\left( {F_{week}^{p} } \right)} \right)$$

24 $$F_{week} = F_{week}^{p} \otimes W_{week}$$

where the fully connected layer has $s_{q}$ neural units.

### Features fusion

This section discusses the fusion method of the three components of local spatio-temporal features, potential daily periodic features, and potential weekly periodic features. As shown in Fig. 5, we denoted these three parts as $F_{close}$, $F_{day}$ and $F_{week}$, respectively. It should be noted that different regions are affected by these three components, but the degree of influence may be different. For some regions, closeness dependence is often particularly significant, while for other regions, long-term dependence is more important. Therefore, when fusing the above three components, we fully considered the different contributions of these three components from historical data.

25 $$F_{res} = w_{rc} \otimes F_{close} + w_{rd} \otimes F_{day} + w_{rw} \otimes F_{week}$$

where ⊗ is the element-wise multiplication, $w_{rc}$, $w_{rd}$ and $w_{rw}$ are learnable parameters that reflect the degrees of the closeness influence, the daily period influence, and the weekly period influence on the predicted target.

Finally, we input $F_{res}$ into a convolutional layer and an activation layer to obtain the taxi OD demand prediction value, $\hat{R}\left( t \right)$ at the time interval t, which is expressed as:

26 $$\hat{R}\left( t \right) = f\left( {Conv\left( {F_{res} ,w_{res} } \right)} \right)$$

where $f$ is the *tanh* activation function, and $w_{res}$ is the learnable parameter.

### Implementation details

We divided Manhattan into a 15 × 5 grid map based on the longitude and latitude. That is, *H* is 15 and *W* is 5. We implemented our model with the Tensorflow-2.5 framework on NVIDIA 3080 GPU. The input to the model consists of eleven historical observations, including five temporal closeness components, three daily periodic components, and three weekly periodic components. That is, *n* is 5, *P* is *3*, and *Q* is 3. In the LSTN module, the number *L* of residual units was set to 2, and all convolution layers in ConvLSTM had 32 filters. For the DayNet and WeekNet sub-network, the convolutional layer had 32 filters and $s_{p} ,s_{q}$ were 3 respectively. For the whole model, the batch size was set to 32, the learning rate was 0.001 in the pre-training model and 0.0001 in the later training, and early stopping on the validation dataset was employed. We used Adam^36^ optimizer for training to minimize Mean Squared Error.

## Conclusions

To address the challenge of taxi service demand prediction in smart cities, we propose a Multi-Sensory Stimulating Attention (MSSA) Model for CTSDP. Like the human perception mechanism, the MSSA model integrates detail perception attention and stimulating variety attention to learn the characteristics of historical demand data at the macro and micro levels. The multiple periodical modules were combined to capture potential spatio-temporal periodicity features from massive taxi service demand data.

Extensive experiments and evaluations were conducted on the taxi travel record dataset in Manhattan, New York. The results showed that the MSSA outperforms the baseline methods. Specifically, compared with the state-of-the-art methods, the MSSA reduced the MAPE and RMSE by 18.29% and 27.66% in the Origin demand forecasting, and 5.26% and 5.30% in the Origin–Destination demand forecasting, respectively. Further analysis showed the effectiveness of multi-sensory stimulating attention and multiple periodicity feature learning.

The MSSA could be applied in smart cities to improve traveling service quality and also help to reduce pollution emissions. In the future, the MSSA could also be extended by learning more periodic patterns and incorporating more context information.

## Data Availability

The yellow taxi trip records data related to this study is accessible using the following links: https://www1.nyc.gov/site/tlc/about/tlc-trip-record-data.page. The meteorological data related to this study is accessible using the following link: https://www.wunderground.com.

## References

[1] Tong Z, Ye F, Yan M, Liu H, Basodi S (2021). A survey on algorithms for intelligent computing and smart city applications. Big Data Min. Anal..

[2] Pang J, Huang Y, Xie Z, Li J, Cai Z (2021). Collaborative city digital twin for the COVID-19 pandemic: A federated learning solution. Tsinghua Sci. Technol..

[3] Lin C (2020). Spatiotemporal congestion-aware path planning toward intelligent transportation systems in software-defined smart city IoT. IEEE Internet Things J..

[4] Riascos A, Mateos JL (2020). Networks and long-range mobility in cities: A study of more than one billion taxi trips in New York City. Sci. Rep..

[5] Wang F (2020). 6G-enabled short-term forecasting for large-scale traffic flow in massive IoT based on time-aware Locality-Sensitive Hashing. IEEE Internet Things J..

[6] Liu Y (2021). An attention-based category-aware GRU model for the next POI recommendation. Int. J. Intell. Syst..

[7] Nikparvar B, Rahman M, Hatami F, Thill J-C (2021). Spatio-temporal prediction of the COVID-19 pandemic in US counties: Modeling with a deep LSTM neural network. Sci. Rep..

[8] Gou Y, Zhang T, Liu J, Wei L, Cui J-H (2020). DeepOcean: A general deep learning framework for spatio-temporal ocean sensing data prediction. IEEE access.

[9] Guo S, Lin Y, Li S, Chen Z, Wan H (2019). Deep spatial–temporal 3D convolutional neural networks for traffic data forecasting. IEEE Trans. Intell. Transp. Syst..

[10] Dong L, Zhang H, Ji Y, Ding Y (2020). Crowd counting by using multi-level density-based spatial information: A Multi-scale CNN framework. Inf. Sci..

[11] Li A, Chen R, Farimani AB, Zhang YJ (2020). Reaction diffusion system prediction based on convolutional neural network. Sci. Rep..

[12] Zhang J (2018). Predicting citywide crowd flows using deep spatio-temporal residual networks. Artif. Intell..

[13] Liu Z, Chen H, Sun X, Chen H (2020). Data-driven real-time online taxi-hailing demand forecasting based on machine learning method. Appl. Sci..

[14] Luo H, Cai J, Zhang K, Xie R, Zheng L (2021). A multi-task deep learning model for short-term taxi demand forecasting considering spatiotemporal dependences. J. Traffic Transp. Eng. (English Edition).

[15] Xu J, Rahmatizadeh R, Bölöni L, Turgut D (2017). Real-time prediction of taxi demand using recurrent neural networks. IEEE Trans. Intell. Transp. Syst..

[16] Liu, Z., Liu, Y., Lyu, C. & Ye, J. Building personalized transportation model for online taxi-hailing demand prediction. *IEEE Trans. Cybern. *(2020). 10.1109/TCYB.2020.3000929 32628608

[17] Wang Y, Xu D, Peng P, Xuan Q, Zhang G (2020). An urban commuters’ OD hybrid prediction method based on big GPS data. Chaos Interdiscip. J. Nonlinear Sci..

[18] Zhao, J., Chen, C., Huang, H. & Xiang, C. Unifying Uber and taxi data via deep models for taxi passenger demand prediction. *Personal Ubiquitous Comput.*, 1–13 (2020).

[19] Rodrigues F, Markou I, Pereira FC (2019). Combining time-series and textual data for taxi demand prediction in event areas: A deep learning approach. Inf. Fusion.

[20] Liu L (2019). Contextualized spatial–temporal network for taxi origin-destination demand prediction. IEEE Trans. Intell. Transp. Syst..

[21] Ke J (2021). Predicting origin-destination ride-sourcing demand with a spatio-temporal encoder-decoder residual multi-graph convolutional network. Transp. Res. Part C Emerg. Technol..

[22] Chen, D., Wang, J. & Xiong, C. Research on origin‐destination travel demand prediction method of inter‐regional online taxi based on SpatialOD‐BiConvLSTM. *IET Intell. Transp. Syst. *(2021).

[23] Rao AR (2018). An oscillatory neural network model that demonstrates the benefits of multisensory learning. Cogn. Neurodyn..

[24] Fordell H, Bodin K, Eklund A, Malm J (2016). RehAtt–scanning training for neglect enhanced by multi-sensory stimulation in Virtual Reality. Top. Stroke Rehabil..

[25] Tibshirani R (2011). Regression shrinkage and selection via the lasso: A retrospective. J. R. Stat. Soc. Ser. B (Stat. Methodol.).

[26] Hutcheson GD (2011). Ordinary Least-Squares Regression.

[27] Chen, T. & Guestrin, C. Xgboost: A scalable tree boosting system. in *Proceedings of the 22nd acm sigkdd International Conference on Knowledge Discovery and Data Mining.* 785–794. 10.1145/2939672.2939785 (2016).

[28] Zhang J, Zheng Y, Qi D (2017). Deep spatio-temporal residual networks for citywide crowd flows prediction. Thirty-first AAAI Conf. Artif. Intell..

[29] *TLC Trip Record Data*, https://www1.nyc.gov/site/tlc/about/tlc-trip-record-data.page (2014).

[30] *Meteorological Data*, https://www.wunderground.com/ (2014).

[31] Yao, H. *et al.* Deep multi-view spatial-temporal network for taxi demand prediction. in *Proceedings of the AAAI Conference on Artificial Intelligence.* (2018).

[32] Zhang K, Zuo W, Chen Y, Meng D, Zhang L (2017). Beyond a gaussian denoiser: Residual learning of deep cnn for image denoising. IEEE Trans. Image Process..

[33] Woo, S., Park, J., Lee, J.-Y. & Kweon, I. S. Cbam: Convolutional block attention module. in *Proceedings of the European conference on computer vision (ECCV).* 3–19. 10.1007/978-3-030-01234-2_1 (2018).

[34] Xingjian, S. *et al.* Convolutional LSTM network: A machine learning approach for precipitation nowcasting. in *Advances in neural information processing systems.* 802–810 (2015).

[35] Yin, X. *et al.* Deep learning on traffic prediction: Methods, analysis and future directions. *IEEE Transactions on Intelligent Transportation Systems* (2021).

[36] Kingma, D. P. & Ba, J. Adam: A method for stochastic optimization. Preprint https://arxiv.org/abs/1412.6980 (2014).

